# Supplementary material for: Bayesian stroke modeling details sex biases in the white matter substrates of aphasia
Source: Commun Biol. 2023 Mar 31;6:354. doi: 10.1038/s42003-023-04733-1 (PMC10066402; doi:10.1038/s42003-023-04733-1)
Supplement: Supplementary file 1 — Supplementary Material [file 42003_2023_4733_MOESM1_ESM.pdf]

Supplementary Online Material

Lesion atom	Women	Men	Sex differences
1	1.31 (1.33)	1.36 (1.33)	*t*=0.59, *p*=0.6
2	0.91 (1.92)	0.85 (1.80)	*t*=-0.68, *p*=0.5
3	0.22 (0.46)	0.21 (0.48)	*t*=-0.25, *p*=0.8
4	0.13 (0.30)	0.14 (0.32)	*t*=0.63, *p*=0.53
5	0.19 (0.39)	0.20 (0.39)	*t*=0.48, *p*=0.63
6	0.16 (0.37)	0.13 (0.33)	*t*=-1.37, *p*=0.17
7	0.70 (0.78)	0.66 (0.77)	*t*=-0.83, *p*=0.41
8	0.30 (0.48)	0.30 (0.48)	*t*=-0.09, *p*=0.93
9	0.18 (0.41)	0.16 (0.37)	*t*=-1.36, *p*=0.17
10	0.34 (0.83)	0.31 (0.78)	*t*=-0.61, *p*=0.54

Supplementary Table 1. Lesion atom expressions across women and men.

The average expression of each lesion atom is depicted as *mean (standard deviation)* for women and men, separately. Sex differences were tested using independent t-tests (t, p-value) or in case of unequal variances using Welch's t-tests (t, p-value).

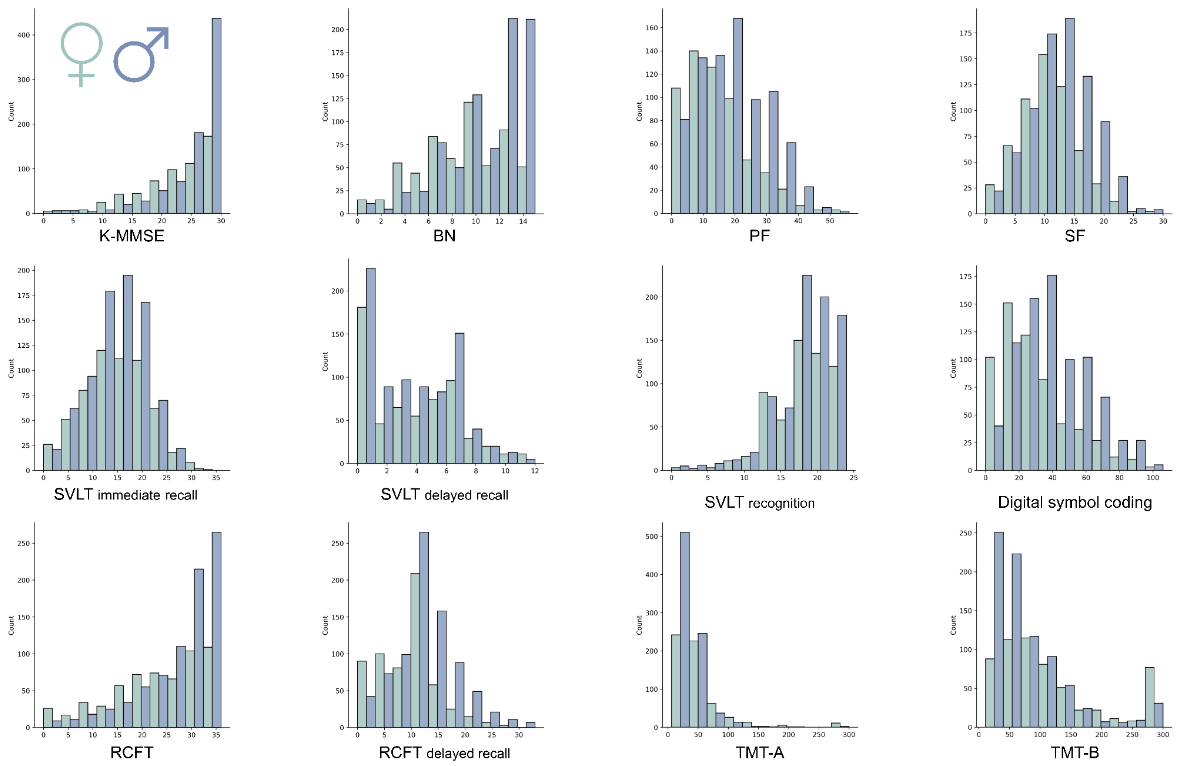

Supplementary Figure 1. Distributions of the neuropsychological performance scores.

For each neuropsychological performance score, the distribution is shown for women (green) and men (blue) separately. Abbreviations: Korean-Mini Mental State Examination: K-MMSE. Boston Naming: BN. Phonemic fluency: PF. Semantic fluency: SF. Seoul-Verbal Learning: SVL. Rey Complex Figure Test: RCFT. Korean-Trail Making Test Version A/B: TMT A/B.

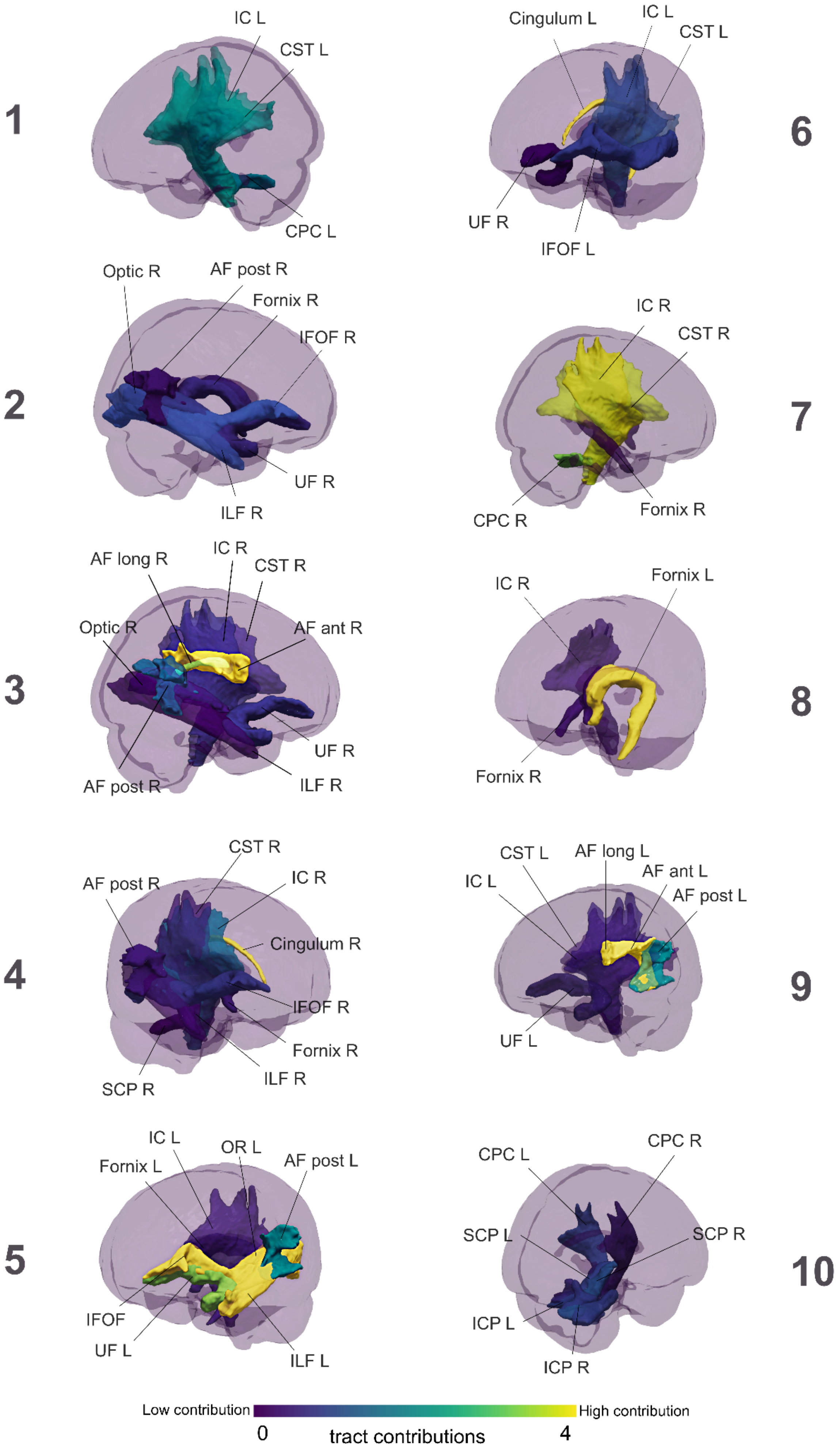
1
IC L
CST L
CPC L
6
Cingulum L
IC L
CST L
UF R
IFOF L
2
Optic R
AF post R
Fornix R
IFOF R
UF R
ILF R
7
IC R
CST R
CPC R
Fornix R
3
AF long R
IC R
CST R
Optic R
AF ant R
UF R
AF post R
ILF R
8
IC R
Fornix L
Fornix R
4
CST R
AF post R
IC R
Cingulum R
IFOF R
Fornix R
ILF R
SCP R
9
CST L
AF long L
AF ant L
IC L
AF post L
UF L
5
IC L
OR L
Fornix L
AF post L
IFOF
UF L
ILF L
10
CPC L
CPC R
SCP L
SCP R
ICP L
ICP R
Low contribution
High contribution
0
tract contributions
4

Supplementary Figure 2. In-depth 3D glass brain renderings of each lesion atom.

Each revealed *lesion atom* (1-10), that is, a unique latent prototypical pattern of white matter stroke lesions, is visualized as 3D glass-brain renderings depicting each underlying tract configuration. The specific white matter tract relevance ranges from high (yellow) to low (dark purple) and corresponds to the complete matrix visualization (factor matrix *W*, cf. Supplementary Figure 3) in Figure 2A. Abbreviations: Arcuate fasciculus: AF. Inferior longitudinal fasciculus: ILF. Uncinate fasciculus: UF. Inferior fronto-occipital fasciculus: IFOF. Cortico-spinal tract: CST. Internal capsule: IC. Cortico-pontine-cerebellar tract: CPC. Inferior and superior cerebellar peduncle: ICP, SCP. Optic radiation: OR. R/L: right/left.

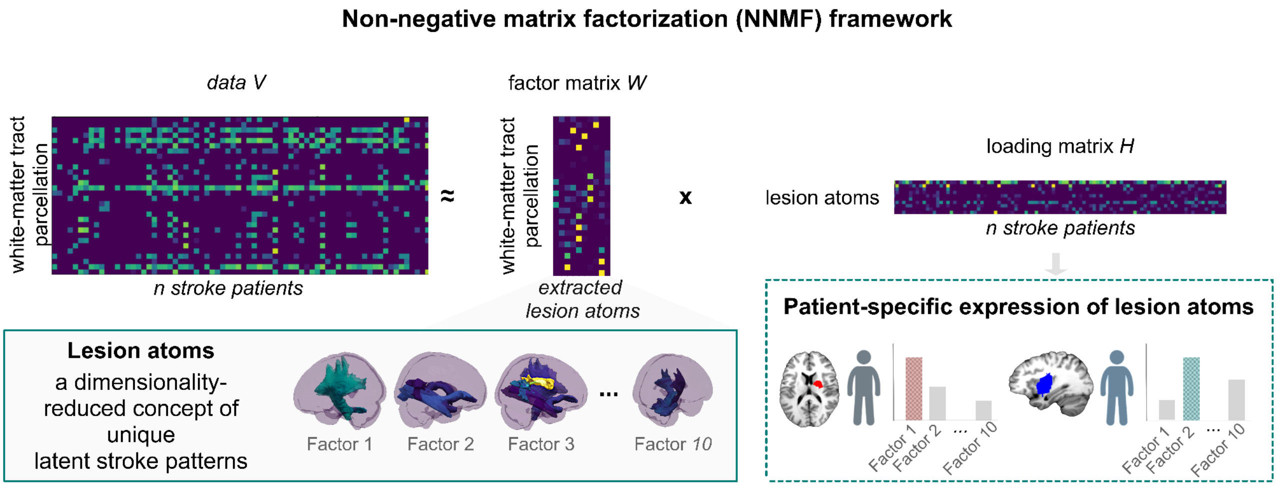

Supplementary Figure 3. Schematic of latent pattern discovery using non-negative matrix factorization (NNMF) for stroke modeling.

We extracted a low-dimensional embedding of the MRI-derived spatial lesion distributions in n=1,401 stroke patients using non-negative matrix factorization (NNMF)[1] as a purely data-driven pattern discovery strategy. The stroke lesion data *V* consisted of tract-by-tract summaries of the individual patient's measures of lesion load parsed by a reference white matter tractography atlas (y-axis)[2,3]. NNMF, as an unsupervised machine learning algorithm, approximates a low-dimensional factor representation *W*. Each ensuing factor, or *lesion atom*[4,5], reflects a unique prototypical latent pattern of stroke-inflicted white matter disconnection. The loading matrix *H* indicates how relevant each lesion atom is to cover or fit (i.e., template-match) an individual patient's overall stroke lesion. Therefore, *H* can be used to extract the patient-specific lesion atom fingerprint. Each stroke lesion distribution found in the individual patient can thus be readily quantified as a parts-based representation of the lesion atoms. For example, patient one depicts a high expression of factor 1, less so of factors 2 and 10. In contrast, patient two's spatial lesion configuration matches factor 2 the most. The patient-specific expression profile, therefore, shows how well each of the discovered lesion archetypes fits the stroke lesion observed in that patient's brain scan.

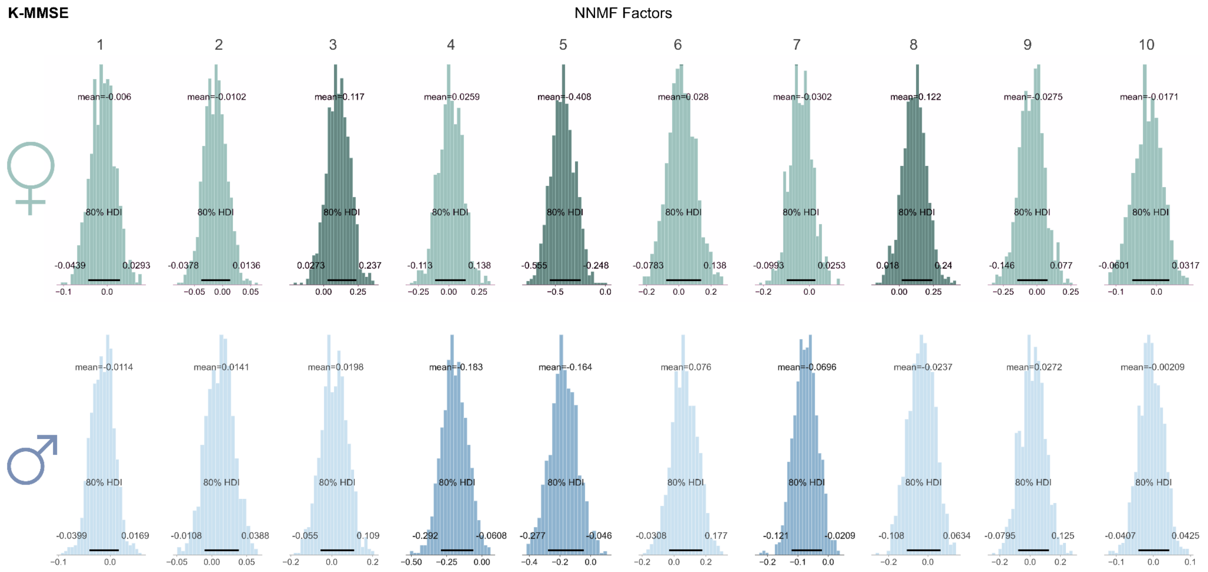

Supplementary Figure 4. K-MMSE Bayesian posteriors

We formed full posterior Bayesian estimates of the parameter distribution to infer the explanatory relevance of each lesion atom (NNMF factors 1-10) for the prediction of Korean-MMSE performance in female (top row, green) and male (bottom row, blue) stroke patients. Lesion atoms were considered relevant (dark shade) if the derived highest density intervals (HDI) of the posterior distribution showed ≥80% certainty (black horizontal line) to be different from zero. Posterior histograms with less intensity marks lower relevance of lesion atoms based on including zero in the corresponding HDI. Abbreviations: Korean-Mini Mental State Examination: K-MMSE. Non-negative matrix factorization: NNMF.

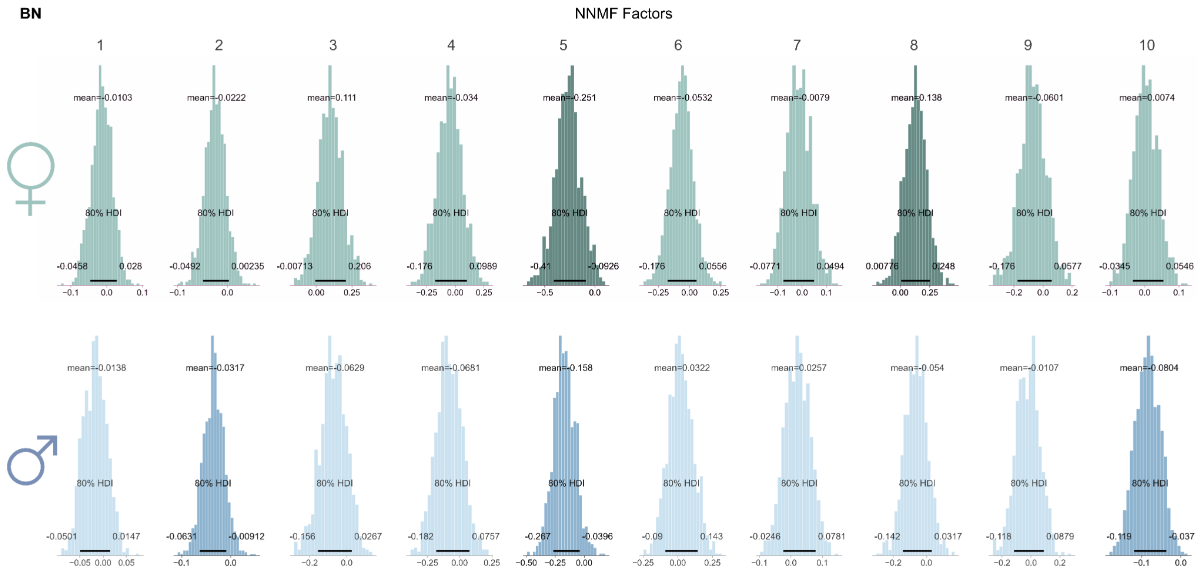

Supplementary Figure 5. BN Bayesian posteriors

We formed full posterior Bayesian estimates of the parameter distribution to infer the explanatory relevance of each lesion atom (NNMF factors 1-10) for the prediction of BN performance in female (top row, green) and male (bottom row, blue) stroke patients. Lesion atoms were considered relevant (dark shade) if the derived highest density intervals (HDI) of the posterior distribution showed ≥80% certainty (black horizontal line) to be different from zero. Posterior histograms with less intensity marks lower relevance of lesion atoms based on including zero in the corresponding HDI. Abbreviations: Boston Naming: BN. Non-negative matrix factorization: NNMF.

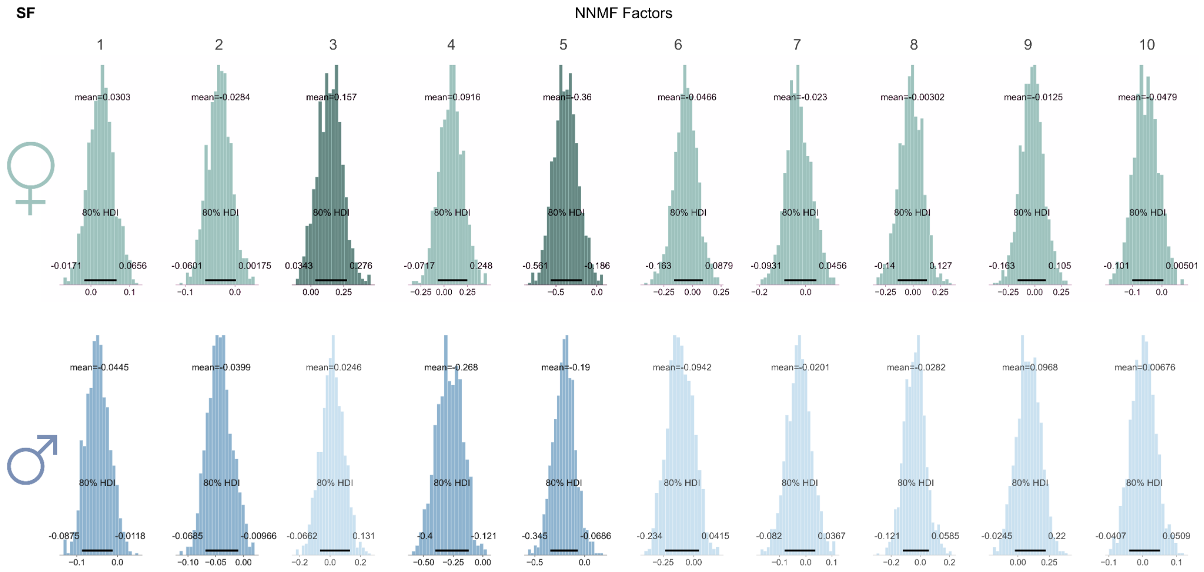

Supplementary Figure 6. SF Bayesian posteriors

We formed full posterior Bayesian estimates of the parameter distribution to infer the explanatory relevance of each lesion atom (NNMF factors 1-10) for the prediction of SF performance in female (top row, green) and male (bottom row, blue) stroke patients. Lesion atoms were considered relevant (dark shade) if the derived highest density intervals (HDI) of the posterior distribution showed ≥80% certainty (black horizontal line) to be different from zero. Posterior histograms with less intensity marks lower relevance of lesion atoms based on including zero in the corresponding HDI. Abbreviations: Semantic fluency: SF. Non-negative matrix factorization: NNMF.

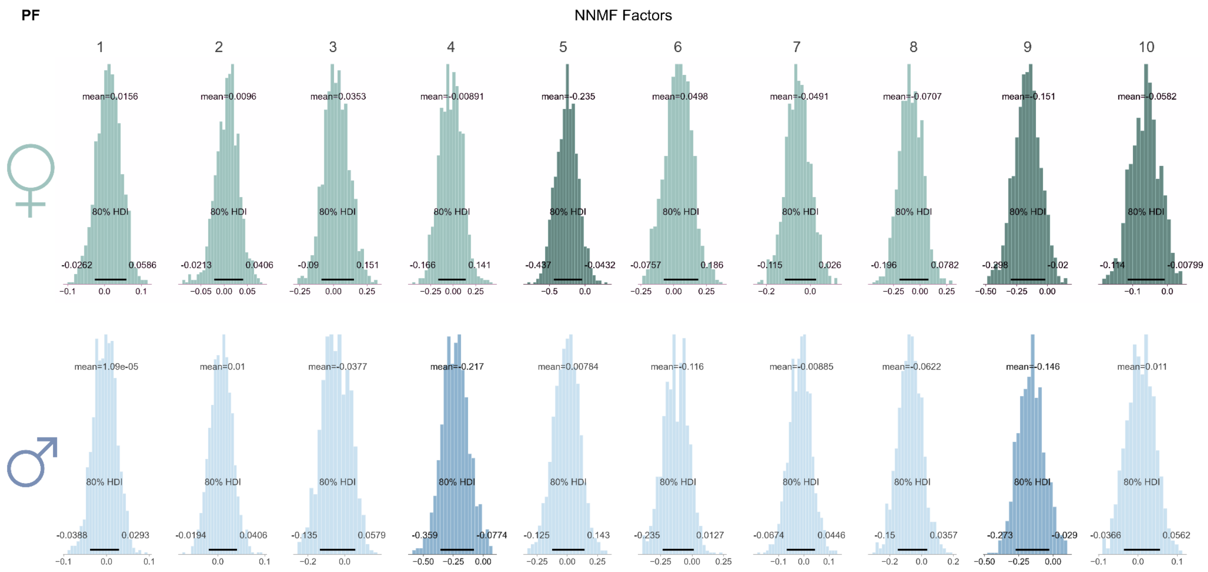

Supplementary Figure 7. PF Bayesian posteriors

We formed full posterior Bayesian estimates of the parameter distribution to infer the explanatory relevance of each lesion atom (NNMF factors 1-10) for the prediction of PF performance in female (top row, green) and male (bottom row, blue) stroke patients. Lesion atoms were considered relevant (dark shade) if the derived highest density intervals (HDI) of the posterior distribution showed ≥80% certainty (black horizontal line) to be different from zero. Posterior histograms with less intensity marks lower relevance of lesion atoms based on including zero in the corresponding HDI. Abbreviations: Phonemic fluency: PF. Non-negative matrix factorization: NNMF.

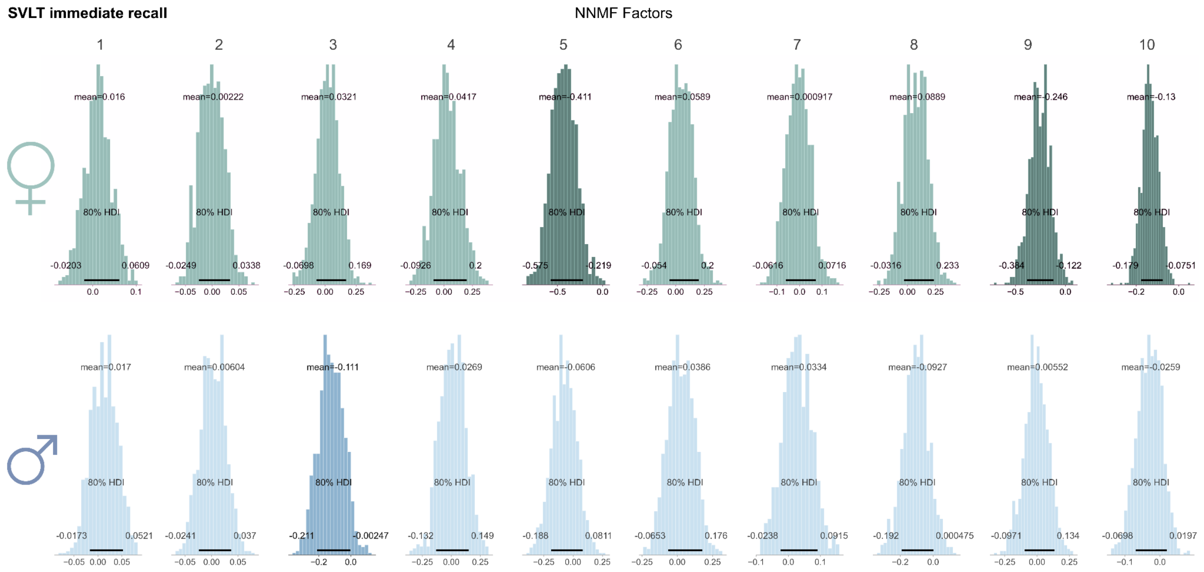

Supplementary Figure 8. SVLT immediate recall Bayesian posteriors

We formed full posterior Bayesian estimates of the parameter distribution to infer the explanatory relevance of each lesion atom (NNMF factors 1-10) for the prediction of SVLT immediate recall performance in female (top row, green) and male (bottom row, blue) stroke patients. Lesion atoms were considered relevant (dark shade) if the derived highest density intervals (HDI) of the posterior distribution showed ≥80% certainty (black horizontal line) to be different from zero. Posterior histograms with less intensity marks lower relevance of lesion atoms based on including zero in the corresponding HDI. Abbreviations: Seoul-Verbal Learning Test: SVLT. Non-negative matrix factorization: NNMF.

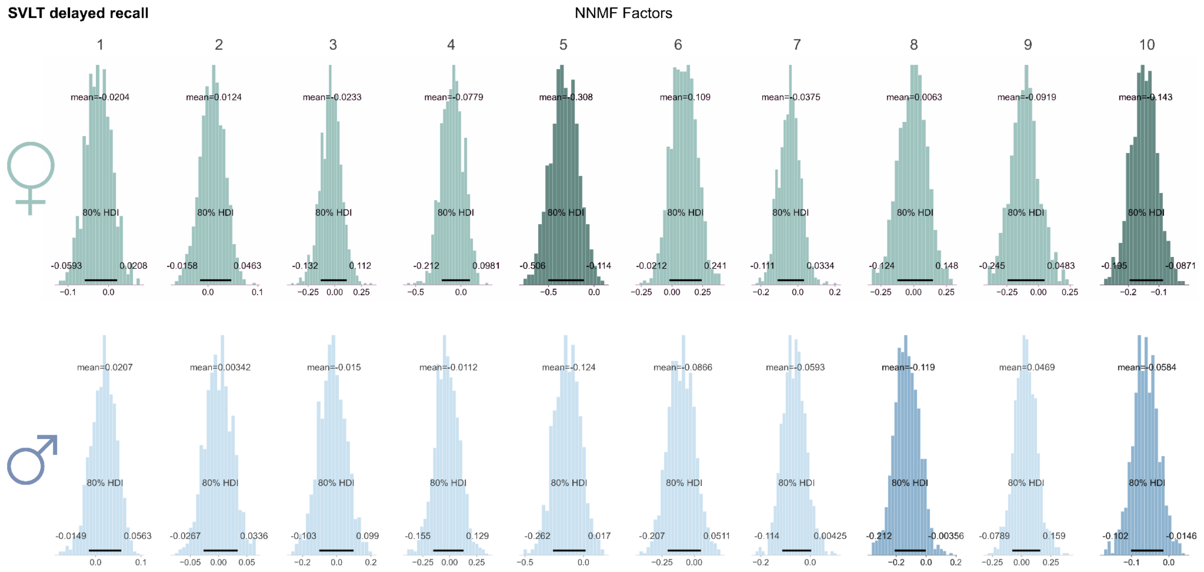

Supplementary Figure 9. SVLT delayed recall Bayesian posteriors

We formed full posterior Bayesian estimates of the parameter distribution to infer the explanatory relevance of each lesion atom (NNMF factors 1-10) for the prediction of SVLT delayed recall performance in female (top row, green) and male (bottom row, blue) stroke patients. Lesion atoms were considered relevant (dark shade) if the derived highest density intervals (HDI) of the posterior distribution showed ≥80% certainty (black horizontal line) to be different from zero. Posterior histograms with less intensity marks lower relevance of lesion atoms based on including zero in the corresponding HDI. Abbreviations: Seoul-Verbal Learning Test: SVLT. Non-negative matrix factorization: NNMF.

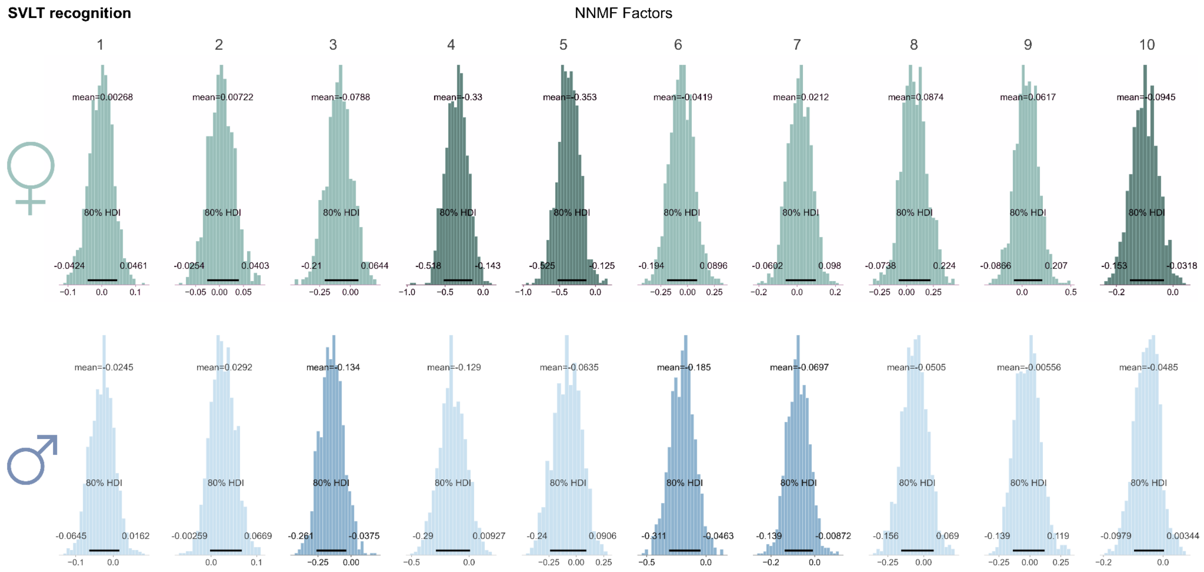

Supplementary Figure 10. SVLT recognition Bayesian posteriors

We formed full posterior Bayesian estimates of the parameter distribution to infer the explanatory relevance of each lesion atom (NNMF factors 1-10) for the prediction of SVLT recognition performance in female (top row, green) and male (bottom row, blue) stroke patients. Lesion atoms were considered relevant (dark shade) if the derived highest density intervals (HDI) of the posterior distribution showed ≥80% certainty (black horizontal line) to be different from zero. Posterior histograms with less intensity marks lower relevance of lesion atoms based on including zero in the corresponding HDI. Abbreviations: Seoul-Verbal Learning Test: SVLT. Non-negative matrix factorization: NNMF.

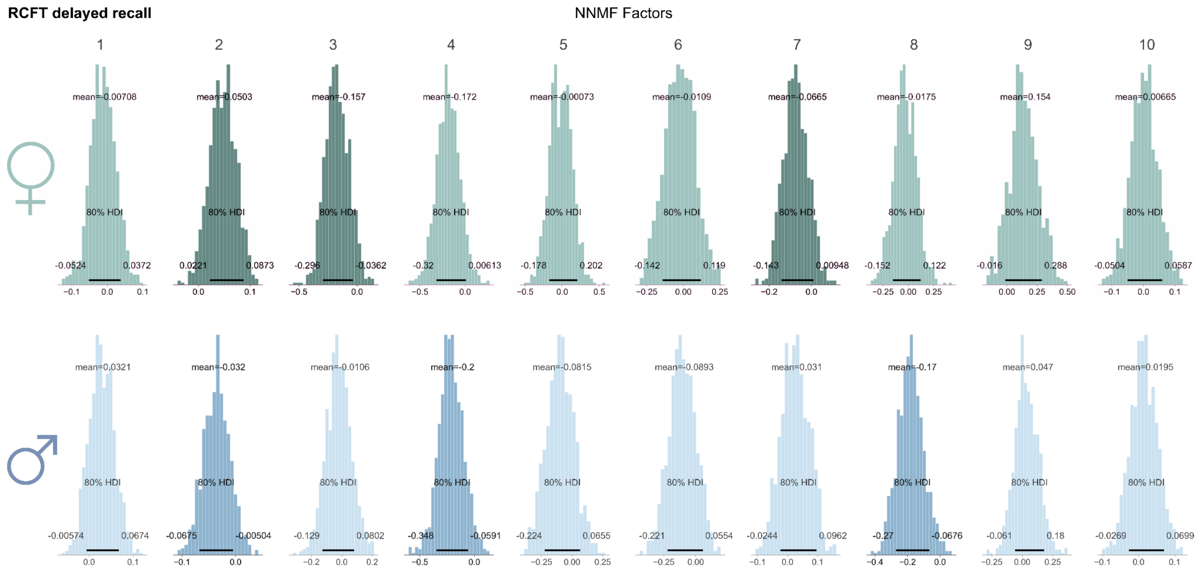

Supplementary Figure 11. RCFT delayed recall Bayesian posteriors

We formed full posterior Bayesian estimates of the parameter distribution to infer the explanatory relevance of each lesion atom (NNMF factors 1-10) for the prediction of RCFT delayed recall performance in female (top row, green) and male (bottom row, blue) stroke patients. Lesion atoms were considered relevant (dark shade) if the derived highest density intervals (HDI) of the posterior distribution showed ≥80% certainty (black horizontal line) to be different from zero. Posterior histograms with less intensity marks lower relevance of lesion atoms based on including zero in the corresponding HDI. Abbreviations: Rey Complex Figure Test: RCFT. Non-negative matrix factorization: NNMF.

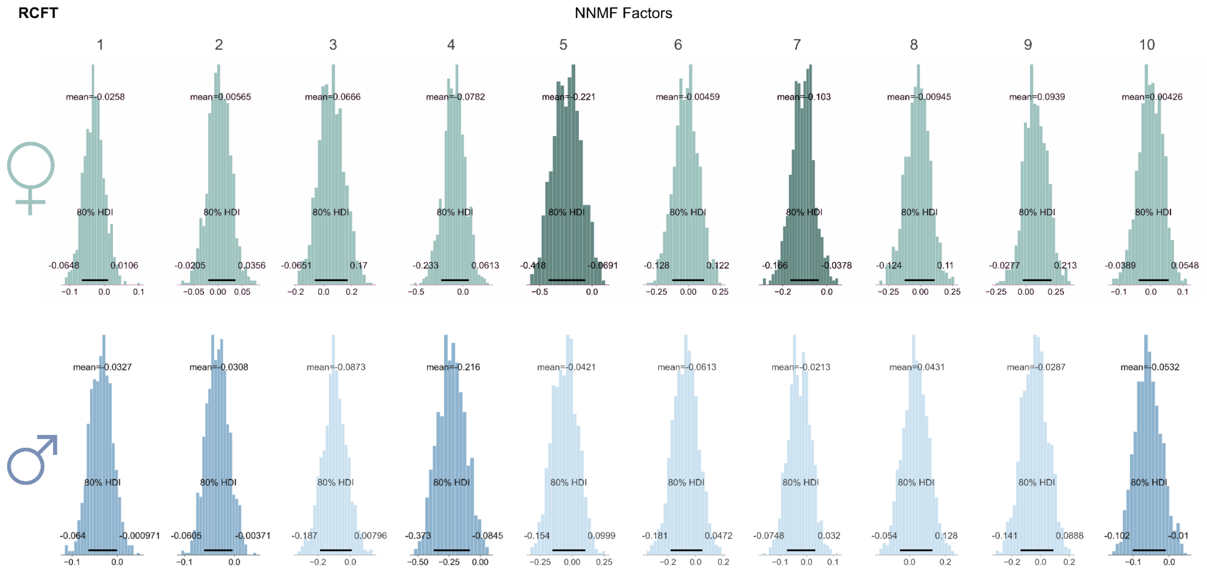

Supplementary Figure 12. RCFT Bayesian posteriors

We formed full posterior Bayesian estimates of the parameter distribution to infer the explanatory relevance of each lesion atom (NNMF factors 1-10) for the prediction of RCFT performance in female (top row, green) and male (bottom row, blue) stroke patients. Lesion atoms were considered relevant (dark shade) if the derived highest density intervals (HDI) of the posterior distribution showed ≥80% certainty (black horizontal line) to be different from zero. Posterior histograms with less intensity marks lower relevance of lesion atoms based on including zero in the corresponding HDI. Abbreviations: Rey Complex Figure Test: RCFT. Non-negative matrix factorization: NNMF.

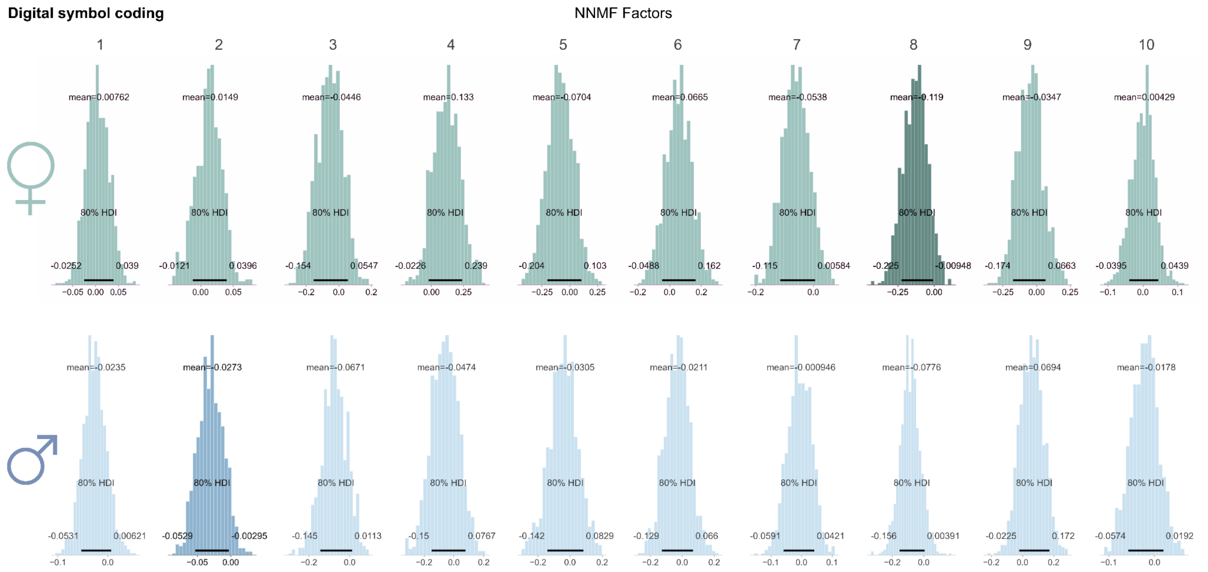

Supplementary Figure 13. Digital symbol coding Bayesian posteriors

We formed full posterior Bayesian estimates of the parameter distribution to infer the explanatory relevance of each lesion atom (NNMF factors 1-10) for the prediction of digital symbol coding performance in female (top row, green) and male (bottom row, blue) stroke patients. Lesion atoms were considered relevant (dark shade) if the derived highest density intervals (HDI) of the posterior distribution showed ≥80% certainty (black horizontal line) to be different from zero. Posterior histograms with less intensity marks lower relevance of lesion atoms based on including zero in the corresponding HDI. Abbreviations: Non-negative matrix factorization: NNMF.

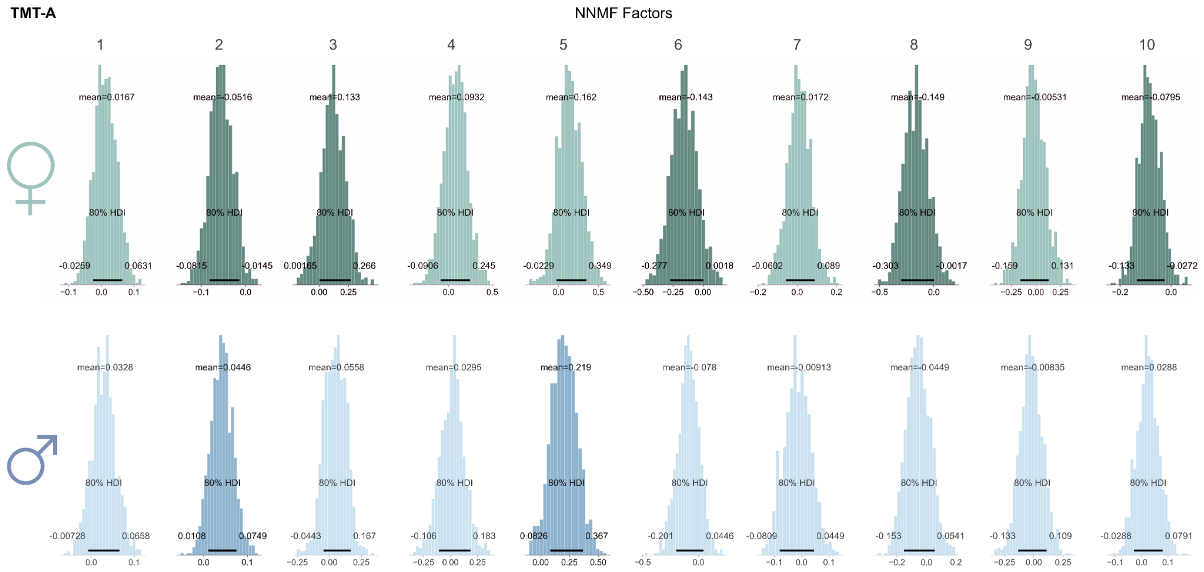

Supplementary Figure 14. TMT-A Bayesian posteriors

We formed full posterior Bayesian estimates of the parameter distribution to infer the explanatory relevance of each lesion atom (NNMF factors 1-10) for the prediction of TMT-A performance in female (top row, green) and male (bottom row, blue) stroke patients. Lesion atoms were considered relevant (dark shade) if the derived highest density intervals (HDI) of the posterior distribution showed ≥80% certainty (black horizontal line) to be different from zero. Posterior histograms with less intensity marks lower relevance of lesion atoms based on including zero in the corresponding HDI. Abbreviations: Korean-Trail Making Test Version A/B: TMT A/B. Non-negative matrix factorization: NNMF.

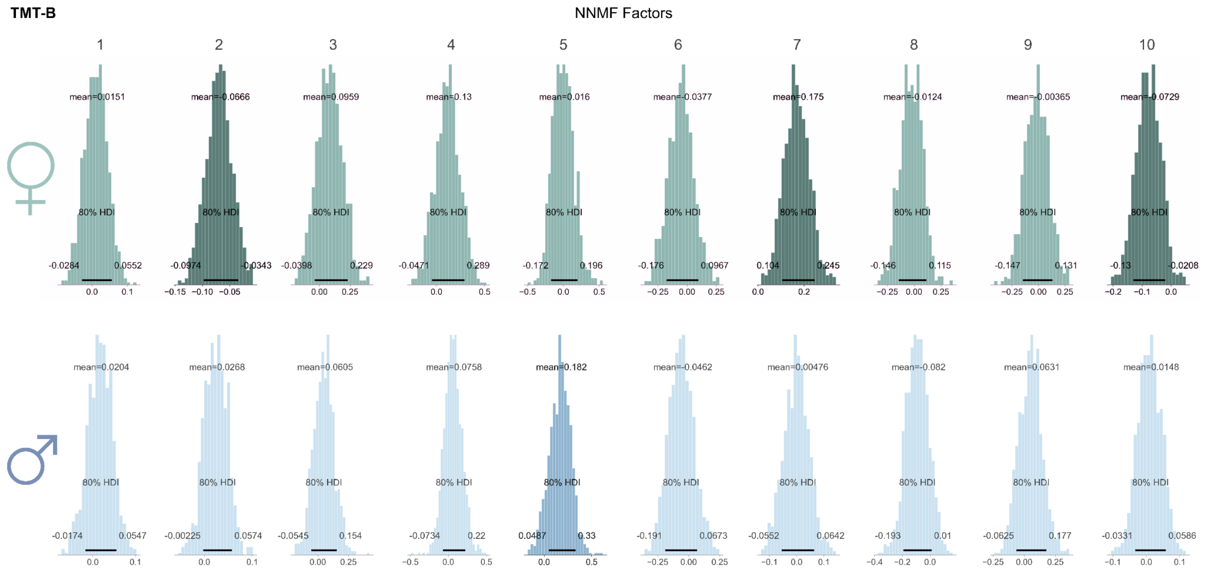

Supplementary Figure 15. TMT-B Bayesian posteriors

We formed full posterior Bayesian estimates of the parameter distribution to infer the explanatory relevance of each lesion atom (NNMF factors 1-10) for the prediction of TMT-B performance in female (top row, green) and male (bottom row, blue) stroke patients. Lesion atoms were considered relevant (dark shade) if the derived highest density intervals (HDI) of the posterior distribution showed ≥80% certainty (black horizontal line) to be different from zero. Posterior histograms with less intensity marks lower relevance of lesion atoms based on including zero in the corresponding HDI. Abbreviations: Korean-Trail Making Test Version A/B: TMT A/B. Non-negative matrix factorization: NNMF.

References

1 Lee DD, Seung HS. Learning the parts of objects by non-negative matrix factorization. *Nature* 1999; **401**: 788–91.

2 Catani M, Thiebaut de Schotten M. A diffusion tensor imaging tractography atlas for virtual in vivo dissections. *Cortex* 2008; **44**: 1105–32.

3 Thiebaut de Schotten M, Ffytche DH, Bizzi A, *et al.* Atlasing location, asymmetry and inter-subject variability of white matter tracts in the human brain with MR diffusion tractography. *Neuroimage* 2011; **54**: 49–59.

4 Bonkhoff AK, Lim J-S, Bae H-J, *et al.* Generative lesion pattern decomposition of cognitive impairment after stroke. *Brain Commun* 2021; **3**: fcab110.

5 Bonkhoff AK, Schirmer MD, Bretzner M, *et al.* Outcome after acute ischemic stroke is linked to sex-specific lesion patterns. *Nat Commun* 2021; **12**: 3289.
