# Supplementary material for: Bayesian stroke modeling details sex biases in the white matter substrates of aphasia
Source: Commun Biol. 2023 Mar 31;6:354. doi: 10.1038/s42003-023-04733-1 (PMC10066402; doi:10.1038/s42003-023-04733-1)
Supplement: Supplementary file 2 — Reporting Summary [file 42003_2023_4733_MOESM2_ESM.pdf]

nature research

Corresponding author(s): D. Bzdok

Last updated by author(s): Feb 20, 2023

Reporting Summary

Nature Research wishes to improve the reproducibility of the work that we publish. This form provides structure for consistency and transparency in reporting. For further information on Nature Research policies, see our Editorial Policies and the Editorial Policy Checklist.

Statistics

For all statistical analyses, confirm that the following items are present in the figure legend, table legend, main text, or Methods section.

n/a	Confirmed	
☐	☒	The exact sample size (*n*) for each experimental group/condition, given as a discrete number and unit of measurement
☐	☒	A statement on whether measurements were taken from distinct samples or whether the same sample was measured repeatedly
☐	☒	The statistical test(s) used AND whether they are one- or two-sided<br>*Only common tests should be described solely by name; describe more complex techniques in the Methods section.*
☐	☒	A description of all covariates tested
☒	☐	A description of any assumptions or corrections, such as tests of normality and adjustment for multiple comparisons
☐	☒	A full description of the statistical parameters including central tendency (e.g. means) or other basic estimates (e.g. regression coefficient) AND variation (e.g. standard deviation) or associated estimates of uncertainty (e.g. confidence intervals)
☐	☒	For null hypothesis testing, the test statistic (e.g. *F*, *t*, *r*) with confidence intervals, effect sizes, degrees of freedom and *P* value noted<br>*Give P values as exact values whenever suitable.*
☐	☒	For Bayesian analysis, information on the choice of priors and Markov chain Monte Carlo settings
☐	☒	For hierarchical and complex designs, identification of the appropriate level for tests and full reporting of outcomes
☐	☒	Estimates of effect sizes (e.g. Cohen's *d*, Pearson's *r*), indicating how they were calculated

Our web collection on statistics for biologists contains articles on many of the points above.

Software and code

Policy information about availability of computer code

Data collection	Bundang and Hallym Vascular Cognitive Impairment cohorts (for details see Kim BJ, Park J-M, Kang K, Lee SJ, Ko Y, Kim JG et al. Case characteristics, hyperacute treatment, and outcome information from the clinical research center for stroke-fifth division registry in South Korea. J Stroke Cerebrovasc Dis 2015; 17: 38–53.). No specific software was used for data collection.
Data analysis	Data analyses were conducted in a Python 3.8.5 (IPython 7.21.0) environment and primarily relied on the packages nilearn (version 0.7.1), sklearn (version 0.24.1), seaborn (version 0.11.1), and PyMC3 (version 3.7). Full code will be accessible after publication.

For manuscripts utilizing custom algorithms or software that are central to the research but not yet described in published literature, software must be made available to editors and reviewers. We strongly encourage code deposition in a community repository (e.g. GitHub). See the Nature Research guidelines for submitting code & software for further information.

Data

Policy information about availability of data

All manuscripts must include a data availability statement. This statement should provide the following information, where applicable:

- Accession codes, unique identifiers, or web links for publicly available datasets
- A list of figures that have associated raw data
- A description of any restrictions on data availability

We analyzed a multi-center stroke registry with 1,401 patients. They were retrospectively selected from the Bundang and Hallym Vascular Cognitive Impairment cohorts, which are prospectively recruited cohorts consisting of patients originally admitted to the Seoul National University Bundang Hospital or Hallym University Sacred Heart Hospital in South Korea between 2007 and 2018 (Kim BJ, Park J-M, Kang K, Lee SJ, Ko Y, Kim JG et al. Case characteristics, hyperacute treatment, and outcome information from the clinical research center for stroke-fifth division registry in South Korea. J Stroke Cerebrovasc Dis 2015; 17: 38–53)

Field-specific reporting

Please select the one below that is the best fit for your research. If you are not sure, read the appropriate sections before making your selection.

☒ Life sciences ☐ Behavioural & social sciences ☐ Ecological, evolutionary & environmental sciences

For a reference copy of the document with all sections, see nature.com/documents/nr-reporting-summary-flat.pdf

Life sciences study design

All studies must disclose on these points even when the disclosure is negative.

Sample size	Largest available multi-center stroke registry; all available subjects that met the inclusion criteria where selected (n=1,401). No power calculation was needed in advance and we used all samples available (see below).
Data exclusions	All patients were eligible for the present study based on the following criteria: (1) availability of brain MRI showing acute tissue infarction in the diffusion-weighted imaging (DWI) and/or fluid-attenuated inversion recovery (FLAIR), (2) successful lesion segmentation and registration, (3) no primary intracerebral hemorrhage, and (4) availability of follow-up data on key demographics and neuropsychological assessment (the 60-min Korean-Vascular Cognitive Impairment Harmonization Standards-Neuropsychology Protocol).
Replication	n/a
Randomization	The analyses in our study use all available subjects that fulfill the criteria described above. Hence there is no equivalent process of randomization that comes into this analysis (this is not a controlled randomised study).
Blinding	For exactly the same reasons (this is not a controlled randomised study), there is no step equivalent to blinding involved.

Reporting for specific materials, systems and methods

We require information from authors about some types of materials, experimental systems and methods used in many studies. Here, indicate whether each material, system or method listed is relevant to your study. If you are not sure if a list item applies to your research, read the appropriate section before selecting a response.

Materials & experimental systems

n/a	Involved in the study	
☒	☐	Antibodies
☒	☐	Eukaryotic cell lines
☒	☐	Palaeontology and archaeology
☒	☐	Animals and other organisms
☐	☒	Human research participants
☒	☐	Clinical data
☒	☐	Dual use research of concern

Methods

n/a	Involved in the study	
☒	☐	ChIP-seq
☒	☐	Flow cytometry
☐	☒	MRI-based neuroimaging

Human research participants

Policy information about studies involving human research participants

Population characteristics	Patients inlcuded in the Bundang and Hallym Vascular Cognitive Impairment cohorts were hospitalized and diagnosed with acute ischemic stroke between 2007 and 2018, for details regarding the cohort see Kim BJ, Park J-M, Kang K, Lee SJ, Ko Y, Kim JG et al. Case characteristics, hyperacute treatment, and outcome information from the clinical research center for stroke-fifth division registry in South Korea. J Stroke Cerebrovasc Dis 2015; 17: 38–53. For details on age, sex, and all other measure cf. Table 1.
Recruitment	Admission of stroke patients, for details see Kim BJ, Park J-M, Kang K, Lee SJ, Ko Y, Kim JG et al. Case characteristics, hyperacute treatment, and outcome information from the clinical research center for stroke-fifth division registry in South Korea. J Stroke Cerebrovasc Dis 2015; 17: 38–53.
Ethics oversight	local institutional review boards of each hospital

Note that full information on the approval of the study protocol must also be provided in the manuscript.

Magnetic resonance imaging

Experimental design

Design type	Clinical scans, no experimental design
Design specifications	Clinical scans, no experimental design
Behavioral performance measures	Clinical scans, no experimental design

Acquisition

Imaging type(s)	T1, T2-weighted spin-echo, FLAIR, and DWI
Field strength	3T
Sequence & imaging parameters	Seoul National University Bundang Hospital<br>FLAIR repetition time: 11,000 ms; echo time: 125 ms; inversion time: 2,800 ms; slice thickness_ 5 mm; intersection gap: 1 mm; matrix: 512 x 512; flip angle 90 degree<br>DWI EPI-spin echo sequence; repetition time: 5,000 ms; echo time: 50 ms; diffusion b-value: 1,000; slice thickness: 5 mm; intersection gap: 1 mm; matrix: 256 x 256; flip angle 90 degree<br><br>Hallym University Sacred Heart Hospital<br>FLAIR repetition time: 11,000 ms; echo time: 125 ms; inversion time: 2,800 ms; slice thickness: 5 mm; matrix: 512 x 512; flip angle 90 degree<br>DWI repetition time: 3,000 ms; echo time: 56 ms; diffusion b-value: 1,000; slice thickness: 5 mm; matrix: 256 x 256; flip angle 90 degree
Area of acquisition	whole brain

Diffusion MRI ☐ Used ☒ Not used

Preprocessing

Preprocessing software	Tissue damage was manually segmented on DWI or less frequently FLAIR images by experienced investigators (A.K.K. and G.A.) relying on in-house developed software based on MeVisLab (MeVis Medical Solutions AG, Bremen, Germany; see Ritter F, Boskamp T, Homeyer A, Laue H, Schwier M, Link F et al. Medical image analysis. IEEE Pulse 2011; 2: 60–70.). Lesion segmentations were checked and potentially refined by two additional experienced raters.
Normalization	Brain scans and their corresponding lesion segmentations were linearly and nonlinearly normalized to Montreal Neurological Institute (MNI-152) reference space employing the publicly available RegLSM image processing pipeline (public code: http://lsm.isi.uu.nl/, for details see Weaver NA, Zhao L, Biesbroek JM, Kuijf HJ, Aben HP, Bae H-J et al. The Meta VCI Map consortium for meta-analyses on strategic lesion locations for vascular cognitive impairment using lesion-symptom mapping: Design and multicenter pilot study. Alzheimers Dement 2019; 11: 310–326.).
Normalization template	Montreal Neurological Institute (MNI-152)
Noise and artifact removal	If there were any visual differences between the original and registered lesion maps during quality control, normalized lesion maps were manually corrected.
Volume censoring	n/a

Statistical modeling & inference

Model type and settings	Bayesian modelling, c.f. Methods section for detailed model description
Effect(s) tested	Sex effects

Specify type of analysis: ☐ Whole brain ☒ ROI-based ☐ Both

Anatomical location(s)	We parsed each patient's lesion fingerprint by summarizing the white-matter damage annotations at the voxel level according to fiber tract definitions from a reference tractography atlas (Catani M, Thiebaut de Schotten M. A diffusion tensor imaging tractography atlas for virtual in vivo dissections. Cortex 2008; 44: 1105–1132; Thiebaut de Schotten M, Ffytche DH, Bizzi A, Dell'Acqua F, Allin M, Walshe M et al. Atlasing location, asymmetry and inter-subject variability of white matter tracts in the human brain with MR diffusion tractography. Neuroimage 2011; 54: 49–59). This widely-used atlas provided spatial definitions of 28 major fiber tracts in MNI standard space.
Statistic type for inference (See Eklund et al. 2016)	Inference was not carried out based on the lesion load parsed by the tractography atlas.

Correction	Samples from the posterior distribution of the model parameters were drawn by the No U-Turn Sampler (NUTS, Hoffman MD, Gelman A, Others. The No-U-Turn sampler: adaptively setting path lengths in Hamiltonian Monte Carlo. J Mach Learn Res 2014; 15: 1593–1623.), a kind of Monte Carlo Markov Chain algorithm (in our setting defaulted to draws=5,000).

Models & analysis

n/a	Involved in the study
☒	☐ Functional and/or effective connectivity
☒	☐ Graph analysis
☐	☒ Multivariate modeling or predictive analysis

Multivariate modeling and predictive analysis	cf. page 20 of the manuscript for detailed Bayesian model structure
